# Imaging ultra thin layers with helium ion microscopy: Utilizing the channeling contrast mechanism

**DOI:** 10.3762/bjnano.3.58

**Published:** 2012-07-12

**Authors:** Gregor Hlawacek, Vasilisa Veligura, Stefan Lorbek, Tijs F Mocking, Antony George, Raoul van Gastel, Harold J W Zandvliet, Bene Poelsema

**Affiliations:** 1 Physics of Interfaces and Nanomaterials, MESA+ Institute for Nanotechnology, University of Twente, PO Box 217, 7500AE Enschede, The Netherlands; 2 Institute for Physics, Montanuniversitaet Leoben, Franz Josef Straße 18, 8700 Leoben, Austria; 3 Inorganic Material Science, MESA+ Institute for Nanotechnology, University of Twente, PO Box 217, 7500AE Enschede, The Netherlands

**Keywords:** channeling, contrast mechanism, helium ion microscopy, ion scattering, thin layers

## Abstract

**Background:** Helium ion microscopy is a new high-performance alternative to classical scanning electron microscopy. It provides superior resolution and high surface sensitivity by using secondary electrons.

**Results:** We report on a new contrast mechanism that extends the high surface sensitivity that is usually achieved in secondary electron images, to backscattered helium images. We demonstrate how thin organic and inorganic layers as well as self-assembled monolayers can be visualized on heavier element substrates by changes in the backscatter yield. Thin layers of light elements on heavy substrates should have a negligible direct influence on backscatter yields. However, using simple geometric calculations of the opaque crystal fraction, the contrast that is observed in the images can be interpreted in terms of changes in the channeling probability.

**Conclusion:** The suppression of ion channeling into crystalline matter by adsorbed thin films provides a new contrast mechanism for HIM. This dechanneling contrast is particularly well suited for the visualization of ultrathin layers of light elements on heavier substrates. Our results also highlight the importance of proper vacuum conditions for channeling-based experimental methods.

## Introduction

The helium ion microscope (HIM) has established itself as a high-performance alternative to the classic scanning electron microscope (SEM). The superior resolution and the outstanding performance on insulating samples are well-known facts [1–2]. While images based on secondary electrons (SE) allow a resolution as good as 0.29 nm [2], backscattered helium (BSHe) images reveal the elemental composition of the specimen. The contrast ratio that can be achieved in both types of images is determined by the chemical composition as well as the crystal orientation. Channeling along low index directions affects SE as well as BSHe images [3–4].

Here, we discuss how channeling can be utilized to gain unexpected contrast in BSHe images on ultrathin surface layers. HIM already provides superior surface sensitivity in SE-based images. The described contrast mechanism for BSHe images extends this capability to backscatter images. We demonstrate how hard-to-visualize thin layers of light elements on top of heavier element substrates can be detected in BSHe mode by clever utilization of channeling into the substrate.

## Experimental

All data presented were recorded on an ultrahigh vacuum (UHV) Orion Plus helium ion microscope from Zeiss [5]. The microscope is equipped with an Everhardt–Thornley (ET) detector to record SE images, and a microchannel plate situated in the beam path below the final lens to record BSHe images. A silicon drift detector to measure the energy of backscattered ions and a Gatan MonoCL4 Elite detector for ionoluminescence complete the setup. The base pressure of 2 × 10^−9^ mbar allows the extended exposure of the same sample area to the He^+^ ion beam. The near absence of hydrocarbons in the sample chamber effectively reduces carbon build-up in the irradiated sample area [5]. Contrast in SE images is primarily based on differences in work function and the yield of SE generation in the region near the surface [6]. As a consequence, HIM has an unprecedented surface sensitivity in SE mode. Together with the high lateral resolution, this permits the routine visualization of thin surface layers [7]. Contrast in BSHe images on the other hand is formed by differences in the backscattering probability of the impinging helium ions. As a rule of thumb these images therefore contain information on the elemental composition of the first 20 nm to 300 nm of the specimen. The backscattering yield has a roughly quadratic dependence on the atomic number (Z) of the target atom. Consequently these images are considered to contain complementary information, namely from the bulk, compared to SE images. However, the obtainable signal intensities will depend on the detector sensitivity. High-resolution images, using the ET detector and a primary energy (PE) of 35 keV, have been recorded for all samples. Since the backscattering yield depends on the scattering cross section, it increases strongly with decreasing primary energy. Therefore, the BSHe data presented here was recorded with lower PEs between 10 keV and 20 keV. This results in a better signal-to-noise ratio for the BSHe images. Patterns of self-assembled monolayers (SAM) were created by using a PDMS stamp and gas-phase silanization. Orthogonal stripes with an identical width of 4 μm of (3-mercaptopropyl)trimethoxysilane (MS, C_6_H_16_O_3_SSi) and triethoxy(1*H*,1*H*,2*H*,2*H*-tridecafluoro-*n*-octyl)silane (PFS, C_14_H_19_F_13_O_3_Si) were formed on the native oxide present on Si{001} wafers [7]. The thickness of the layers corresponds to the length of the molecules, which are 7 Å and 11 Å for MS and PFS, respectively. *para*-Sexiphenyl (6P) thin films were grown on Si{001} wafers covered by a native oxide in an UHV system with a base pressure of 1 × 10^−10^ mbar. Prior to thin-film growth the substrate was flashed to 500 °C. 6P was deposited at room temperature from a Knudsen cell [8–9]. For the formation of cobalt nanoclusters, an atomically clean Ge{001} substrate was obtained by prolonged 800 eV Ar^+^ ion sputtering followed by annealing of the sample through resistive heating at 1100 K. Several monolayers of Co were evaporated by resistively heating a tungsten wire wrapped with a pure Co (99.995%) wire. During evaporation the sample was kept at room temperature. Afterwards it was annealed at 600 K for 8 min and for a shorter period of 4 min at 700 K. Before insertion into the HIM the sample was briefly exposed to air during which time a thin oxide layer most likely formed [10].

Angle-dependent projections of the silicon crystal lattice, to obtain measures for the backscattering probability, were calculated by using a simple geometric model of the crystal slab. For some of these calculations a graphene-like carbon overlayer was added to the silicon slab. Atom radii were fixed to 0.42 Å and 0.30 Å for silicon and carbon, respectively. Lattice constants of 5.43 Å and 2.46 Å were used for silicon and carbon, respectively. To speed up the calculations the thickness of the crystal slab was restricted to 24 layers or six unit cells. This thickness equals 3.3 nm, which corresponds roughly to the escape depth of the SE in the HIM [6]. The crystal slab was rotated and tilted with respect to the [001] direction and the projected blocked area fraction (opacity) was calculated [3] for the area of one unit cell, or in other words eight neighboring channels. Due to the mismatch in unit-cell size, the positions of the carbon adatoms were different in these eight channels. To average over many possible configurations for the overlayer atoms, the adlayer was shifted across 25 different positions relative to the bulk. SRIM [11] calculations to obtain measures for the backscatter probability and the range of the helium particles were performed with SRIM-2008 and the quick Kinchin–Pease formalism [11–12]. To ensure a sound statistical result 1 × 10^5^ He ions of the selected energy were traced in appropriately thick slabs of the bulk material.

## Results

### Thin organic layers

**Figure 1:**
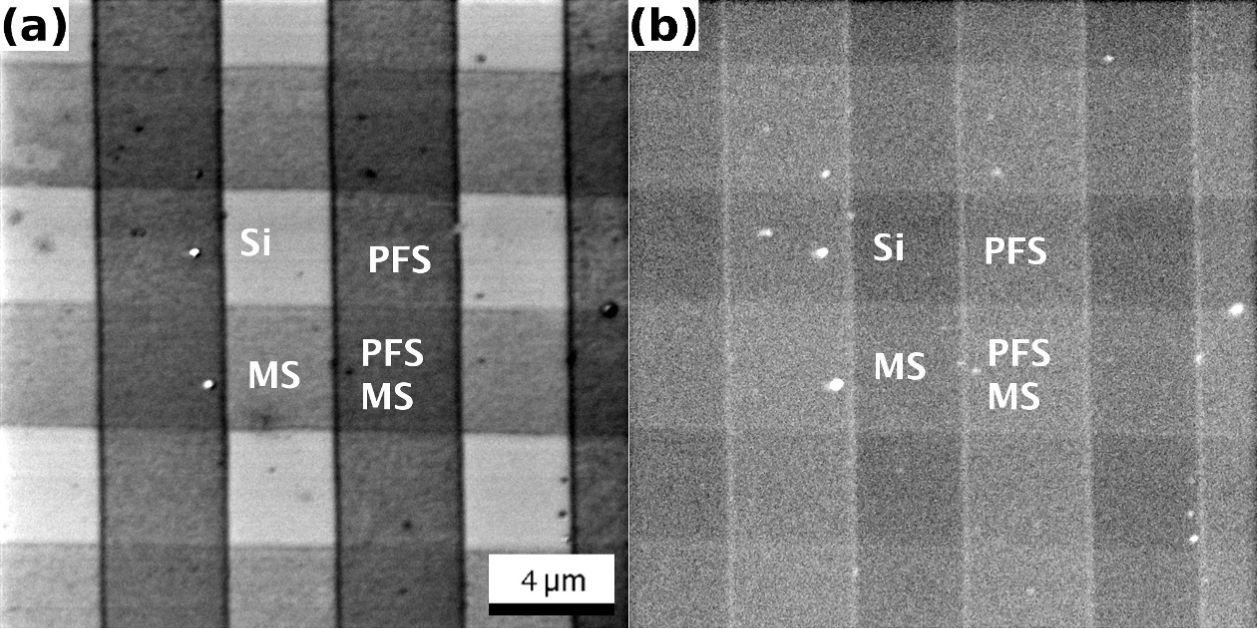
HIM images with a FoV of 20 μm of thin organic layers on Si{001}. Data was recorded with a PE of 15 keV, and an ion dose of 2.46 × 10^16^ cm^−2^. (a) ET image of stripes of PFS (vertical) and MS (horizontal). The different areas and their termination are indicated. (b) BSHe image recorded simultaneously with (a). The different surface terminations can be distinguished.

In Figure 1 HIM images of a network of two SAMs, namely MS and PFS, are presented. The images were recorded with a PE of 15 keV and an ion dose of 2.46 × 10^16^ cm^−2^ under normal beam incidence. Figure 1a was obtained by using the ET detector. SEs in HIM originate from near surface regions. The characteristic escape depth of SEs in carbon is 1 nm [6]. The high contrast between the different patches, and the high lateral resolution, are a result of this characteristic of the SEs in HIM. All the SEs contributing to the different contrast patches are generated under identical conditions, nearly exclusively within a thin surface layer of the relevant material (SiO_2_, PFS, or MS). As a consequence of the identical strip width for PFS and MS strips, we do not know a priori which stripe is which. However, we assign the bright structureless areas to the uncovered SiO_2_/Si substrate. It is understood that because of the relatively low work function of SiO_2_, these areas are brightest. The work functions of PFS and MS are 6.6 eV and 5.3 eV [13], respectively. The value for PFS was extrapolated from a shorter fluorinated alkanethiol [14], and should be treated as an estimate. We can therefore identify the medium light-gray areas below and above the Si patches as being MS-covered. The medium dark areas to the left and the right of the Si patches are covered by the higher-work-function PFS layer. The remaining square is covered by an unknown mixture of both, MS and PFS. A clear statement on the work function or the contrast mechanism for this remaining patch is therefore difficult. Figure 1b shows the simultaneously recorded BSHe image. Interestingly, the SAMs are not only discernible but can also be distinguished. In addition, small details at the edge of the vertical SAM stripes are clearly visible. The relative average backscattered He yields with respect to SiO_2_/Si (BSHe yield: 1) are 1.58 and 1.45 for PFS and MS, respectively. We will discuss the underlying contrast mechanisms below; however, we first highlight two more examples of ultrathin surface structures that are made visible in BSHe images.

**Figure 2:**
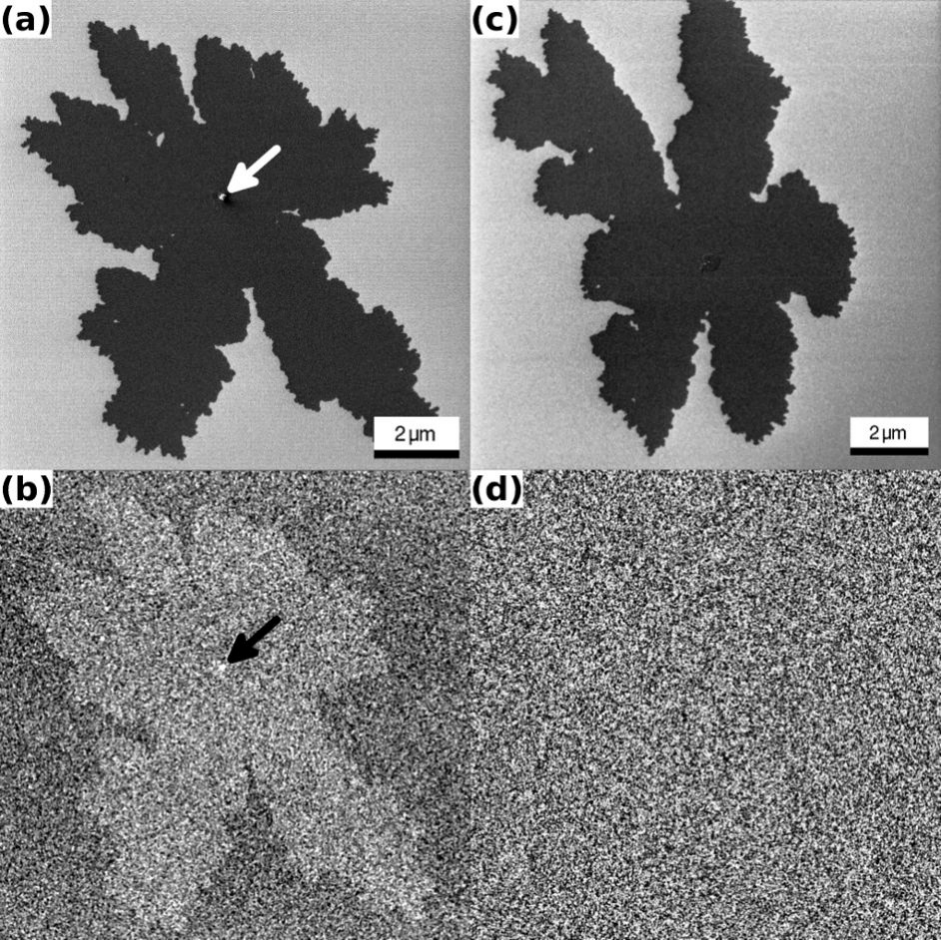
HIM images of single-layer 6P islands on Si{001}, recorded with PE of 20 keV and an ion dose of 3.21 × 10^15^ cm^−2^. (a) ET image with a FoV of 11 μm recorded under normal incidence. The island and a small second-layer island (bright spot in the center, marked by an arrow) can be seen. (b) Corresponding BSHe image. The island and the second-layer island (marked by an arrow) can be seen. (c) ET image with a FoV of 12 μm recorded under identical conditions as in (a,b) but with an incidence angle of 10°. The island can be seen clearly. (d) In the corresponding BSHe image the island is invisible.

In Figure 2 HIM images of single-layer high (≈2.6 nm) 6P islands on native-oxide-covered Si{001} wafers are shown [8–9]. Figure 2a is an ET image of such an island. The FoV is 11 μm, the PE was 20 keV and an ion dose of 3.21 × 10^15^ cm^−2^ was used. The ramified shape of the island (dark) is clearly visible against the bright silicon substrate. Figure 2b is the simultaneously recorded BSHe image. The shape of the island (bright) can easily be distinguished against the darker background of the silicon substrate. Figure 2c shows a different island recorded with a sample tilt of 10°, but otherwise under unchanged conditions. The corresponding BSHe image presented in Figure 2d does not, however, show a signature of the island. We note that the overall gray level in Figure 2d is found to be higher than for the bare silicon in Figure 2b and close to the one of the 6P island in Figure 2b.

### Inorganic nanocrystals

**Figure 3:**
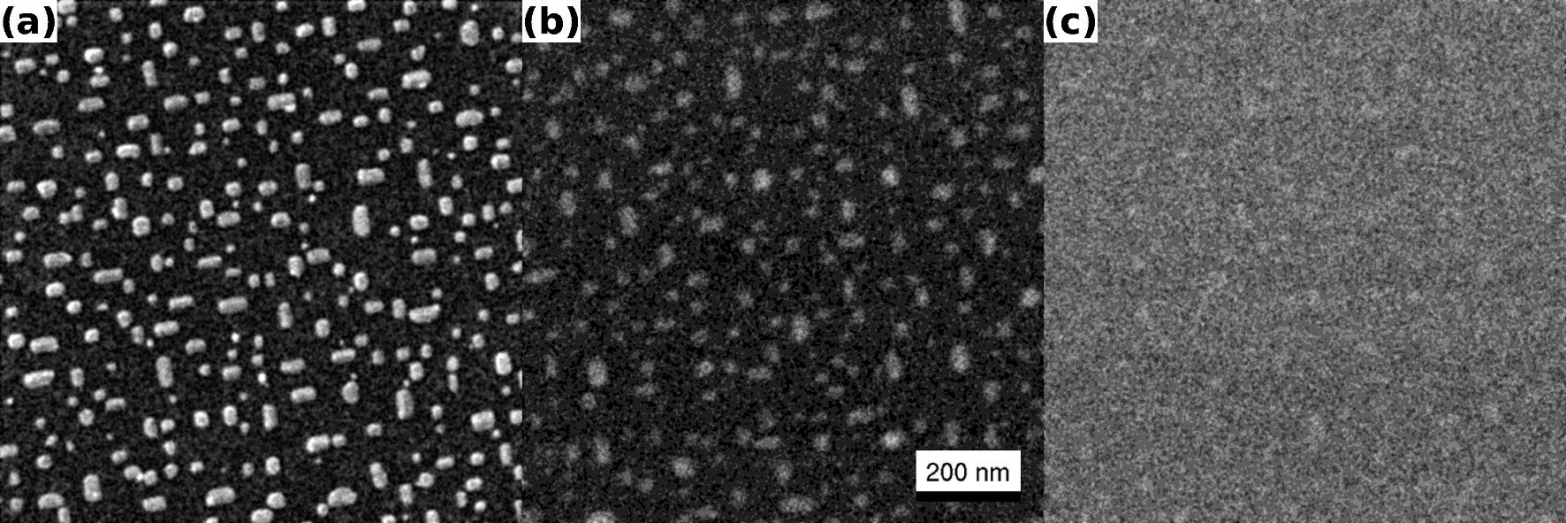
Co-containing nanocrystals on Ge{001} (FoV: 1 μm) (a) High-resolution ET image obtained with a PE of 34.6 keV and an ion dose of 1.05 × 10^16^ cm^−2^. Aligned Co-containing nanocrystals are visible. The average extent of the crystals is between 10 nm and 60 nm laterally with a height of around 5 nm. (b) BSHe image obtained under normal incident with a PE of 10 keV and an ion dose of 1.05 × 10^16^ cm^−2^. The Co-containing nanocrystals are clearly visible. (c) BSHe image recorded under identical conditions as used in (b) but with an incident angle of 10°. Reprinted from [10], copyright (2012) with permission from Elsevier.

As a third example of the same contrast mechanism, we present selected results of a study dealing with the growth of Co islands on Ge{001} [10]. Figure 3a shows epitaxially aligned Co islands with average sizes between 10 nm and 60 nm. The aspect ratio varies from 1 up to approximately 3 with 1.54 and 1.20 being the mean and the mode of the distribution, respectively. The height of the nanocrystals was found to vary between 4 nm and 7 nm [10]. Figure 3b is a 1 μm FoV BSHe image recorded with a PE of 10 keV and an ion dose of 1.05 × 10^16^ cm^−2^. The islands and the different lateral shapes are clearly visible. In contrast, in Figure 3c, which was recorded under identical conditions but with a sample tilt of 10°, the islands are hardly discernible.

## Discussion

As we have seen above, BSHe images can be used to obtain information on ultrathin surface structures on crystalline substrates. In this context, the last part of the previous sentence is important. We now discuss the role of channeling in the underlying crystalline substrate for obtaining the BSHe images presented above. In all three experiments a thin layer of a lighter element(s) was deposited on top of a heavier substrate. Different to the ET images, in which SEs are generated in regions near the surface, the backscattering of He is a bulk effect. For a layer of heavy elements on a lighter substrate one expects an increased BSHe yield for the following two reasons: (1) The heavier element has a larger cross section and will therefore add to the BSHe yield; (2) the adlayer decreases the energy of the primary beam, thereby increasing the backscatter probability and reducing the range of helium in the material. The increased scattering will lead earlier, in terms of energy and depth, to hard collisions with large scattering angles and result in a larger deviation from the initial particle trajectory. We will discuss this in more depth in the next paragraph. For the present case in which a light adlayer (either carbon or cobalt) covers a heavier substrate (silicon or germanium), (1) does not play a significant role and (2) will be weak in general.

To underline the above statement, SRIM calculations were used to obtain a generic view of the expected processes. Artificial silicon samples with a thickness of 1 μm and a 10 nm adlayer of either heavy (Pb) or light (Li) elements, and without an adlayer were compared. 1 × 10^5^ He^+^ ions with a PE of 35 keV under normal incidence were used to perform the calculation. The results are summarized in Table 1. As expected, the backscattering yield for Pb/Si (1.9%) is higher by a factor of two compared to the other two combinations (0.9% for both cases). While the light adlayer does not affect the lateral range and straggle of the He, the heavy adlayer induces an 8% larger lateral range and a 10% increased lateral straggle. Here, straggle is defined in accordance with the SRIM software to be the square root of the second moment of the range distribution [11]. Although these values represent averages that are dominated by the ions stopped deep in the sample, a comparable relative change will occur closer to the surface for the helium particles that will eventually be backscattered. This will have a negative influence on the lateral resolution that can be achieved in BSHe images. SE images will not be affected since the SEs are generated in the first few nanometers of the sample where the beam is still sharply focused.

**Table 1:** Scattering process dependence on adlayer material as obtained by SRIM. For each adlayer/sample combination the number of backscattered helium atoms and the longitudinal and radial ion ranges (in Å) are given. 1 × 10^5^ He ions with a PE of 35 keV under normal incidence were used in the calculation.

sample	BSHe	direction	range [Å]	straggle [Å]

Pb/Si	1863	long.	3095	952
		rad.	1567	782
Li/Si	881	long.	3261	918
		rad.	1443	712
Si	979	long.	3190	917
		rad.	1446	714

For the case of the SAMs on SiO_2_ (Figure 1), SRIM calculations were performed to estimate the backscatter probability from the different layers. Bulk samples of SiO_2_, MS, and PFS were created and exposed to 5 × 10^5^ He^+^ ions with an energy of 15 keV. The backscattering probabilities obtained are 1.73, 0.69, and 0.71% for SiO_2_, MS, and PFS, respectively. In this model calculation SiO_2_ has the highest backscattering probability and should appear brightest in BSHe images. Keeping in mind the considerations mentioned in the previous paragraph and the calculated backscatter probabilities, no additional contribution is to be expected from the BS- or PFS-covered areas. However, a detailed analysis of the image data reveals that, relative to SiO_2_/Si, the backscatter probabilities are higher by a factor of 1.45 and 1.58 for MS and PFS, respectively.

**Figure 4:**
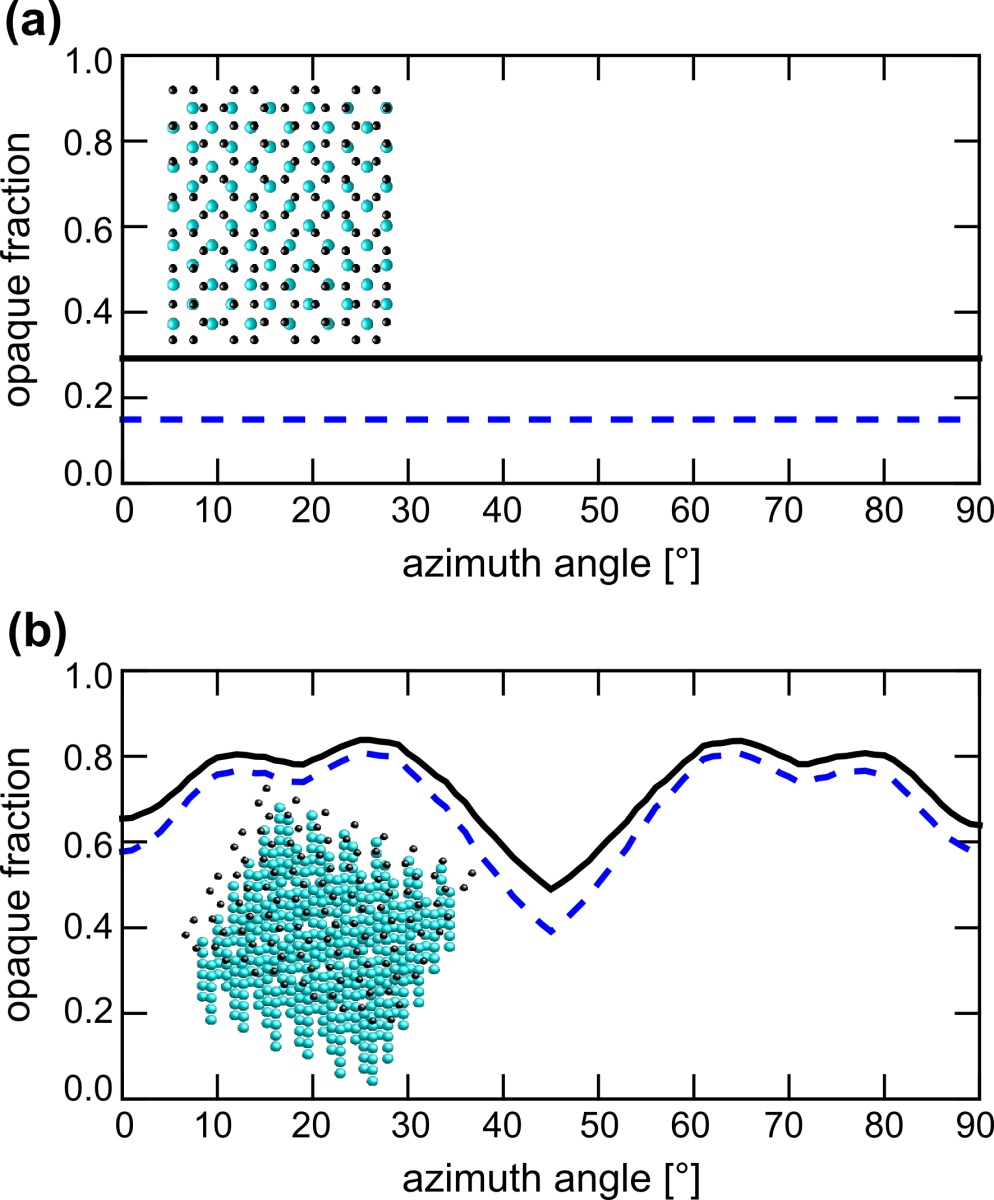
Simulation of dechanneling contrast for clean and carbon-covered Si. The graphs show the opaque fraction of the projected crystal lattice. Blue dashed lines are obtained for a clean Si(001) crystal, whereas the black lines are obtained with a thin carbon layer added. (a) Normal incidence. The opaque projected area fraction is 15 and 29% for the clean and carbon-covered surface, respectively. There is no azimuthal dependence for this incident angle. (b) The same calculation but for a 10° sample tilt. The average opaque projected area fractions are 68 and 73% for the clean and carbon covered surface, respectively. A clear dependence on the azimuthal angle exists.

As we have demonstrated in Figure 2 and Figure 3, the polar angle of the incident He^+^ beam is critical for the contrast in BSHe images. In Figure 4 we show the result of calculations of the opaque area fraction for a silicon {001} crystal. The graphs show the opaque fraction of the crystal, which is proportional to the backscattering yield. For normal beam incidence (Figure 4a) 15% of the area is blocked (blue dashed line). Adding a single carbon overlayer increases the opaque fraction to 29% (dark solid line). At normal incidence, this corresponds to an increase in the blocked fraction by 66%, independent of the azimuthal angle. Tilting the incident beam with respect to the surface normal increases the overall backscattering probability, but reduces the expected contrast ratio between a clean Si crystal and one that is covered by a single adlayer. The increased yield of backscattered He is evident by comparing Figure 2b and Figure 2d. The BSHe yield has increased substantially for the uncovered surface areas. The expected contrast depends on the azimuthal angle and varies between 26% and 4% with a mean value of 8% for a 10° beam tilt. The insets in Figure 4 show the model crystal slabs with carbon adlayer that were used, illustrating the reduced transparency for the tilted cases. Despite the simplicity of this model it nicely confirms the physics involved in the decrease in contrast between areas with and without an adlayer when the sample is tilted. For thicker adlayers this effect is going to be more pronounced because the channels in the underlying crystal are more effectively blocked. In fact, the amount of backscattered He due to the thin 6P adlayer in Figure 2b is comparable to the amount for the uncovered, but tilted, area in Figure 2d.

The results presented in Figure 1, Figure 2, and Figure 3 are based on the angle-dependent channeling probability described above. In all three cases the surface is covered by a native oxide. The thickness and nature of this oxide layer is unknown. We assume that it is of the order of 2 nm and amorphous. This will cause a partial blocking of the underlying channels in Si{001} or Ge{001}. However, the effectiveness of the dechanneling will depend on the thickness of the overlayer. A local increase in thickness of the amorphous overlayer will increase the contrast, because more He is backscattered. This can be seen in the organic overlayer, in particular for the rims of the vertical stripes of PFS in Figure 1(b). The edges of the stripes are thicker [7], and this leads to an increased chance for an ion to be deviated from the initial trajectory. Consequently, this results in more backscattering of He due to the enhanced dechanneling. A similar effect can be observed for the small second-layer island on top of the 6P island in Figure 2a and Figure 2b.

## Conclusion

Besides the possibility to obtain crystallographic information, channeling can also be used to obtain information on ultrathin organic and inorganic layers. We demonstrated that even a thin layer of submonolayer coverage can be detected in BSHe images. The enhanced backscattering is a result of changes in the channeling probability and does not depend on the mass of the participating film or bulk atoms. As an unanticipated result, light adlayers on heavy substrates can be imaged. We emphasize that this contrast mechanism is purely based on changes in the crystallography of the sample. Apart from the detection of ultrathin adlayers, this mechanism therefore also has the potential to reveal crystal defects, such as dislocations or clusters of interstitial atoms. In fact the contrast mechanism has been successfully applied to the Co on Ge system. In this case the new contrast mechanism reveals the different structural nature of the Co-containing nanocrystals on top of the Ge{001} substrate. The fact that the crystallites can only be seen under incident beam angles that allow channeling into Ge{001} is a sign of their different structural properties. The Co in the crystallites influences the position of the atoms sufficiently to block the channels in the covered part of the Ge{001} surface. This independently supports the scanning tunneling spectroscopy results, which show that the crystals are cobaltgermanides [10]. Due to the small size of these crystallites, this information is difficult to obtain by other techniques such as diffraction methods or transmission electron microscopy.

Finally, we wish to stress the point that this is a clear hint for the importance of good vacuum conditions during HIM measurements. From our geometrical-projection-based calculation, we conclude that just a single monolayer of carbon can result in a 66% contrast loss. This not only affects the general performance of the imaging technique but will in particular affect channeling-based contrast mechanisms.
